# Polycystic ovary syndrome: Identification of novel and hub biomarkers in the autophagy-associated mRNA-miRNA-lncRNA network

**DOI:** 10.3389/fendo.2022.1032064

**Published:** 2022-11-29

**Authors:** Jiayu Huang, Baoyi Huang, Yanxiang Kong, Yazhu Yang, Chengzi Tian, Lin Chen, Yan Liao, Lin Ma

**Affiliations:** Laboratory for Assisted Reproduction and Reproductive Genetics, the Reproductive Medical Center, the Seventh Affiliated Hospital of Sun Yat-sen University, Shenzhen, Guangdong, China

**Keywords:** polycystic ovary syndrome, autophagy, autophagy-related genes, weighted gene co-expression network analysis, K-means algorithm

## Abstract

**Introduction:**

Polycystic ovary syndrome (PCOS) is a common metabolic and endocrine disorder prevalent among women of reproductive age. Recent studies show that autophagy participated in the pathogenesis of PCOS, including anovulation, hyperandrogenism, and metabolic disturbances. This study was designed to screen autophagy-related genes (ATGs) that may play a pivotal role in PCOS, providing potential biomarkers and identifying new molecular subgroups for therapeutic intervention.

**Methods:**

Gene expression profiles of the PCOS and control samples were obtained from the publicly available Gene Expression Omnibus database. The gene lists of ATGs from databases were integrated. Then, the weighted gene co-expression network analysis was conducted to obtain functional modules and construct a multifactorial co-expression network. Gene Ontology and KEGG pathway enrichment analyses were performed for further exploration of ATG's function in the key modules. Differentially expressed ATGs were identified and validated in external datasets with the Limma R package. To provide guidance on PCOS phenotyping, the dysfunction module consists of a co-expression network mapped to PCOS patients. A PCOS-Autophagy-related co-expression network was established using Cytoscape, followed by identifying molecular subgroups using the Limma R package. ps. RNA-sequencing analysis was used to confirm the differential expression of hub ATGs, and the diagnostic value of hub ATGs was assessed by receiver operating characteristic curve analysis.

**Results:**

Three modules (Brown, Turquoise, and Green) in GSE8157, three modules (Blue, Red, and Green) in GSE43264, and four modules (Blue, Green, Black, and Yellow) in GSE106724 were identified to be PCOS-related by WGCNA analysis. 29 ATGs were found to be the hub genes that strongly correlated with PCOS. These hub ATGs were mainly enriched in autophagy-related functions and pathways such as autophagy, endocytosis, apoptosis, and mTOR signaling pathways. The mRNA-miRNA-lncRNA multifactorial network was successfully constructed. And three new molecular subgroups were identified via the K-means algorithm.

**Discussion:**

We provide a novel insight into the mechanisms behind autophagy in PCOS. BRCA1, LDLR, MAP1B, hsa-miR-92b-3p, hsa-miR-20b-5p, and NEAT1 might play a considerably important role in PCOS dysfunction. As a result, new potential biomarkers can be evaluated for use in PCOS diagnosis and treatment in the future.

## Introduction

Polycystic ovary syndrome (PCOS) is a metabolic and endocrine disorder that commonly affects women of fertile age. As a heterogeneous disorder, PCOS is defined by various signs and symptoms including polycystic ovary morphology, androgen excess, and ovulatory dysfunction (1). It affects approximately 6–20% of women worldwide and leads to a marked reduction in life quality (2, 3). PCOS not only leads to complications such as anovulation and infertility but also tends to diseases associated with metabolic and reproductive deformities including diabetes, obesity, endometrial cancer, and cardiovascular disease (4).

Autophagy, a self-eating process of cellular material, serves as a dynamic degrading and recycling system producing new building blocks and energy that are essential for survival, differentiation, development, and homeostasis (5, 6). Autophagy is characterized by cytoplasmic vacuolization, autophagosome formation, and clearance of material *via* the lysosome (5). Initially identified through genetic screens in yeast, Autophagy is carried out by an evolutionarily conserved molecular process encoded by Autophagy-related genes (ATG) (7, 8). As a cell death mechanism, autophagy derives from its role in developmentally programmed cell death (9). In metabolic disorders such as T2DM and obesity, autophagy might contribute to regulating cellular metabolism (10).

Evidence from previous research signifies the role of autophagy in PCOS development. The inhibition and activation of autophagy appear to alter PCOS progression. Impaired autophagy is involved in declining granulosa cell population, disturbed folliculogenesis, and metabolic disturbance. In the ovary, autophagy is necessary for the development of follicles. Under normal circumstances, autophagy played an important role in governing follicular development including oocyte development, follicular growth and differentiation, follicular atresia, and the reproductive cycle (11, 12). Dysfunction of autophagy in ovarian follicular cells may reduce oocytes quality thus leading to female infertility (13). Recent studies have suggested a prominent role played by autophagy in the pathophysiological mechanisms associated with PCOS such as anovulation, disordered folliculogenesis, hyperandrogenism, and metabolic disturbances (14). Autophagy is involved in aberrant folliculogenesis in the PCOS ovary. Autophagy causes follicular atresia by inducing granulosa cell death in the pre-antral follicle (13, 15). Autophagy is associated with anovulation in PCOS. The fundamental cause of anovulation associated with PCOS is aberrant folliculogenesis at the initial stages. Autophagy plays a crucial role in metabolic abnormalities in PCOS (14). Autophagy governs the metabolic response in various metabolic organs such as adipose tissue (16), ovary (17), skeletal muscles (18), liver (19), and heart (20). Thus, targeting autophagy may provide a network between ovarian physiology, hyperandrogenism, insulin resistance, obesity, and endocrine disturbances concerning PCOS. However, detailed knowledge in this area is lacking and the underlying mechanism remains to be further explored.

Weighted gene co-expression network analysis (WGCNA), is one of the most important and widely used methods of bioinformatic systems (21). As an R package for weighted correlation network analysis, WGCNA is usually applied to describe the correlation patterns among genes *via* microarray specimens (22). The WGCNA could not only retrieve a cluster of genes with similar biological functions but also might connect modules to external clinical features. Therefore, relevant functional networks constructed by WGCNA could be applied to identify specific biomarkers and new molecules. K-means, a traditional clustering method using Lloyd heuristic, has played an important role in multiple downstream tasks of machine learning (23). The k-means is popular due to its simplicity, efficiency and stable performance. Thus, k-means is recommended to cluster data into different groups based on similarity/distance between data points (24).

In this study, we reveal new molecular subgroups of ATGs that may be useful for PCOS. WGCNA identified new molecular modules and hub genes of autophagy in PCOS. BRCA1, LDLR, MAP1B, and NEAT1 were proven to be potential diagnostic biomarkers and validated by external datasets. Also, we constructed a multifactorial network of PSOS phenotypes associated with hub ATGs. This work provides critical insights into how PCOS should be diagnosed early and treated promptly.

## Materials and methods

### Microarray data

Multiple high-throughput datasets of PCOS were downloaded from the Gene Expression Omnibus (GEO) database (http://ncbi.nlm.nih.gov/geo/). To explore the function of ATGs in PCOS-associated anovulation, aberrant folliculogenesis, and endocrine and metabolic disturbances, we utilized samples of granulosa cells, adipose tissue, and skeletal muscles as target-organs. We retrieved multiple GEO series, including mRNA-lncRNA expression profiles, a) GSE106724: 12 samples of ovarian granulosa cells from normal control (n=4) and women with PCOS (n=8); b) GSE43264: 15 samples of subcutaneous adipose tissue from normal control (n=7) and women with PCOS (n=8); c) GSE8157: 43 samples of skeletal muscle from healthy control(n=13), obese women with PCOS (n=10) and obese women with PCOS before/after pioglitazone treatment (30 mg/day for 16 weeks) (n=10). The miRNA expression profile, GSE72274: 10 samples of ovarian granulosa cells of PCOS patients (n = 5) and normal people (n = 5). The external databases were used to further confirm the significance and accuracy of our results, including a) GSE95728: 14 samples of ovarian granulosa cells from normal control (n=7) and women with PCOS (n=7); b) GSE2508: 20 samples of subcutaneous adipose tissue from lean women (n=10) and obese women (n=10); c) GSE6798: 29 samples of skeletal muscle from healthy control(n=13) and women with PCOS(n=16). The available anthropometric and endocrine parameters of the samples from different GEOs were provided in **Supplemental Table 1**. We extracted ATGs from databases including AUTOPHAGY DATABASE (http://www.tanpaku.org/autophagy/index.html), Human Autophagy Database (http://www.autophagy.lu/index.html), HAMdb (http://hamdb.scbdd.com/), GSEA (https://www.gsea-msigdb.org/gsea/index.jsp) and NCBI (https://www.ncbi.nlm.nih.gov/). A total of 2,209 ATGs were identified in the datasets (**Supplemental Table 2**).

### Differential expression analysis

After downloading the original data from GEO, it was normalized *via* the “affy” package in R software. Then, significance analysis of microarrays was applied to analyze each cohort (PCOS and healthy people) to discover differential expression genes, respectively. This process was accomplished *via* limma in R software. In this method, we considered that a p-value<0.05 was statistically significant.

A heat map was created using the “pheatmap” package in R.

The Linear Models for Microarray Data (limma) Package for R were used for the identification of differential expression genes.

### Weighted gene co-expression network analysis

To construct the gene co-expression network and identify the functional modules, we also used WGCNA to analyze the differential expressed genes in women with PCOS. Sample outliers were identified *via* hierarchical clustering analysis performed using the Principal Component Analysis method. Then WGCNA algorithm was applied to calculate the ATGs correlation and construct the gene co-expression modules.

A connection weight is allocated to each gene pair and soft thresholds are used instead of binary information in traditional methods. Step-by-step network con construction was applied with an appropriate soft threshold power (R^2 =^ 0.75) and a minimum module size of 30. Dendrograms were generated by calculating the topological overlap distance from the adjacency matrix, then clustered using average linkage hierarchical clustering. Using a dynamic tree-cut strategy, modules were identified by clustering genes in a hierarchical manner. Then, gene co-expression modules were constructed using WGCNA, and gene information was extracted from each module. A total of 1,892, 1,727, and 1,476 ATGs were mapped to the expression profile of the GSE8157, GSE43264, and GSE106724 samples, respectively. And several functional modules with highly correlated ATGs were identified.

### Enrichment analysis of key modules

Enrichment analysis was conducted to analyze, identify, and interpret varying biological functions in the related modules. The Gene Ontology (GO) annotation analysis (http://geneontology.org/y) was applied to identify the possible molecular function and visualize the potential biological meaning. The biology process terms with a p-value<0.05 under the hypergeometric test were considered statistically significant. Kyoto Encyclopedia of Genes and Genomes (KEGG) annotation analysis (http://www.genome.ad.jp/kegg/) was used to analyze the potential functions of these genes. The data were visualized and analyzed using the ClusterProfiler and ggplot2 R packages (p-value Cutoff<0.05).

### Identification of Hub ATGs

According to the obtained gene significance scores, the numbers of genes in key modules were arranged in sequence from high to low. Hub ATGs represents the top-ranked genes in the inter-modular connectivity, which are centrally located in their respective module and can reflect their characteristics. Compared with the other genes in the network, the hub genes of key modules have a greater biological significance.

Differential expression analyses were performed between the PCOS group and control group *via* the limma R package, on the GSE106724, GSE43264, and GSE8157 datasets (p < 0.05). We applied WGCNA to identify the ATGs correlation in each module. Then Cytoscape (version 3.9.1; http://www.cytoscape.org/) was used to predict hub ATGs (25).

To validate our results, the differential expression of the hub genes was validated in three independent datasets respectively, including GES6798, GSE2508, and GSE95728.

### Dysfunction modules in the PCOS phenotyping

To evaluate the potential function of key modules in PCOS phenotyping, the machine learning assay-K-means, unsupervised clustering method was applied to classify all PCOS samples in an independent database GSE95728 (23). The receiver operating characteristic (ROC) curve analysis curve was used to assess the discriminative accuracy of hub ATGs in key prediction models. And the area under the receiver operating characteristic curve (AUC) was applied to describe the probability that predicted risks following previous strategies (26). The accuracy of the score was assessed by calculating the ROC curve and analyzing the area under the AUC.

### New multifactorial network visualization

We extracted the PCOS-autophagy-related mRNA-miRNA-lncRNA network. We mapping the differential expression genes (DEGs) into the modules. miRNAs and lncRNAs were, respectively, predicted with the miRNA database (v3.0; http://mirwalk.umm.uni-heidelberg.de/) and starBase databases (v2.0; http://starbase.sysu.edu.cn/). The interaction axes were selected according to the overlapped miRNAs that interacted with DEGs. The limma R package was applied to analyze the differential expression of miRNA and lncRNA in GSE72274 and GSE95728. The Cytoscape software was used for visualization of the mRNA-miRNA-LncRNA regulatory network.

### RNA extraction and RNA-sequencing analysis

Ovarian granulosa cell samples were collected from patients at the Center for Reproductive Medicine, the Seventh Affiliated Hospital of Sun Yat-sen University, Shenzhen, Guangdong, China. The diagnosis of PCOS was based on the Revised Rotterdam Diagnostic Criteria, which requires the presence of at least two of the following criteria for a PCOS diagnosis: a) oligo-ovulation and/or anovulation; b) clinical and/or biochemical signs of hyperandrogenism; and c) polycystic ovaries (27). The inclusion criteria for control women were as follows: a) regular menstrual cycles (25–35 days), b) no endocrine abnormalities, and c) normal ovarian morphology confirmed by ultrasound. Total RNA of granulosa cells from 6 samples, including 3 women with PCOS and 3 healthy people, with matched age and BMI (**Supplemental Table 1**) was extracted using the TRIzol reagent (Life Technologies, CA, USA). rRNA was removed using the MGIEasy rRNA Removal Kit. The library was sequenced on a DNBSEQ platform and 100 bp paired-end reads were generated after library preparation. RNA-sequencing (RNA-seq) raw read sequences were aligned against human genome assembly GRCh38.p13 by HISAT2 (v2.0.4). featureCounts (v1.5.0-p3) was used to count the reads numbers mapped to each gene. Differential expression analysis of two groups was performed using the limma R package. Genes with an adjusted P-value <0.05 were designated as deferentially expressed. The gene expression profile is available at National Genomics Data Center(NGDC) database (https://ngdc.cncb.ac.cn/) (BioProject ID: PRJCA012180).

## Results

### Identification of key modules

**Figure 1:**
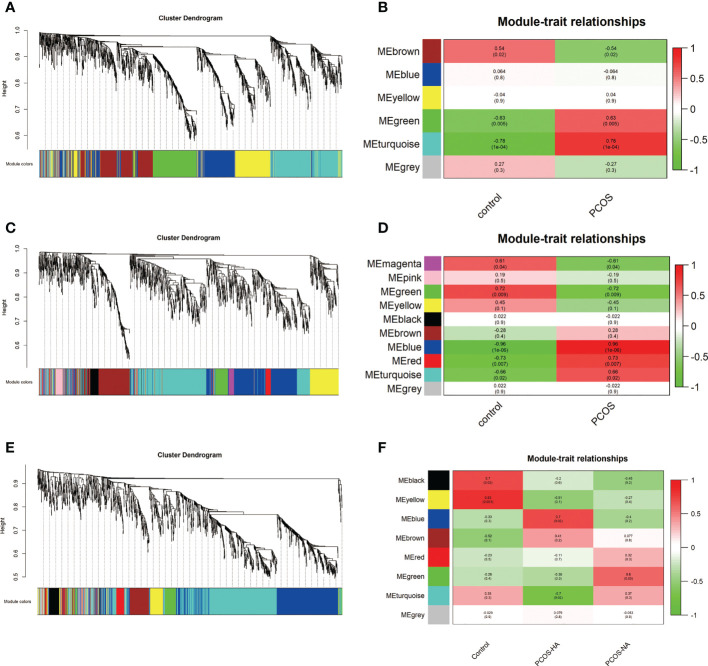
The Weighted gene co-expression network analysis (WGCNA) of key modules involved in polycystic ovary syndrome (PCOS)-associated autophagy. **(A, C, E)** Hierarchical cluster tree showing co-expression modules based on WGCNA in GSE8157 **(A)**, GSE43264 **(C)**,and GSE106724 **(E)**. The color row underneath the dendrogram shows the module assignment determined by the Dynamic Tree Cut. **(B, D, F)** Correlation between key modules and clinical traits in GSE8157 **(B)**, GSE43264 **(D)** and GSE106724 **(F)**. Y-axis shows the modules. The first row of each module is the R2 value and the closer R2 is to 1, the stronger the correlation is. The second row of each module is the p-value and p < 0.01 was considered statistically significant.

Based on GSE8157, GSE43264, and GSE106724 datasets, WGCNA was applied to detect the PCOS-autophagy-related modules. According to the average linkage hierarchical clustering, we identified six modules in GSE8157 datasets (indicated by the colors Blue, Brown, Yellow, Green, Turquoise, and Grey) (**Figures 1A, B**), ten modules in GSE43264 datasets (indicated by the colors of Magenta, Pink, Green, Yellow, Black, Brown, Blue, Red, Turquoise, and Grey) (**Figures 1C, D**) and eight modules in GSE106724 datasets (indicated by the colors of Black, Yellow, Blue, Brown, Red, Green, Turquoise, and Grey) (**Figures 1E, F**, see **Supplemental Table 3** for the genes in each module).

The analysis of the relationship between the gene co-expression modules and clinical traits resulted in several findings. The modules might be preserved because of their Z‐score > 5 and p-value < 0.05. And key modules were further considered to be crucial for PCOS because there were relatively many differentials expressed genes enriched in them.

The Brown, Turquoise, and Green modules were identified as key modules in GSE8157 datasets. The genes in the Green, Turquoise module were positively correlated with the clinical traits of the PCOS group (R2 = 0.63, p value= 0.005; R2 = 0.78, p value= 1e-04). While the Brown modules were negatively correlated with the clinical features of the PCOS group (**Figure 1B**). In GSE43264 datasets, Blue and Red modules were further considered to be crucial for the PCOS group (R2 = 0.96, p value= 1e-07; R2 = 0.73, p value=0.007). The Green module was positively correlated with the control group (R2 = 0.72, p value=0.009) (**Figure 1D**). The Blue and Green modules were identified as key modules and positively correlated with the PCOS-HA (Hyperandrogenism) and NA(Normal androgen) groups in GSE106724 datasets (R2 = 0.60, p value= 0.05; R2 = 0.70, p value= 0.02). The Black and Yellow modules were negatively correlated with the both PCOS group (R2 = 0.70, p value= 0.02; R2 = 0.83, p value= 0.001) (**Figure 1F**).

**Figure 2:**
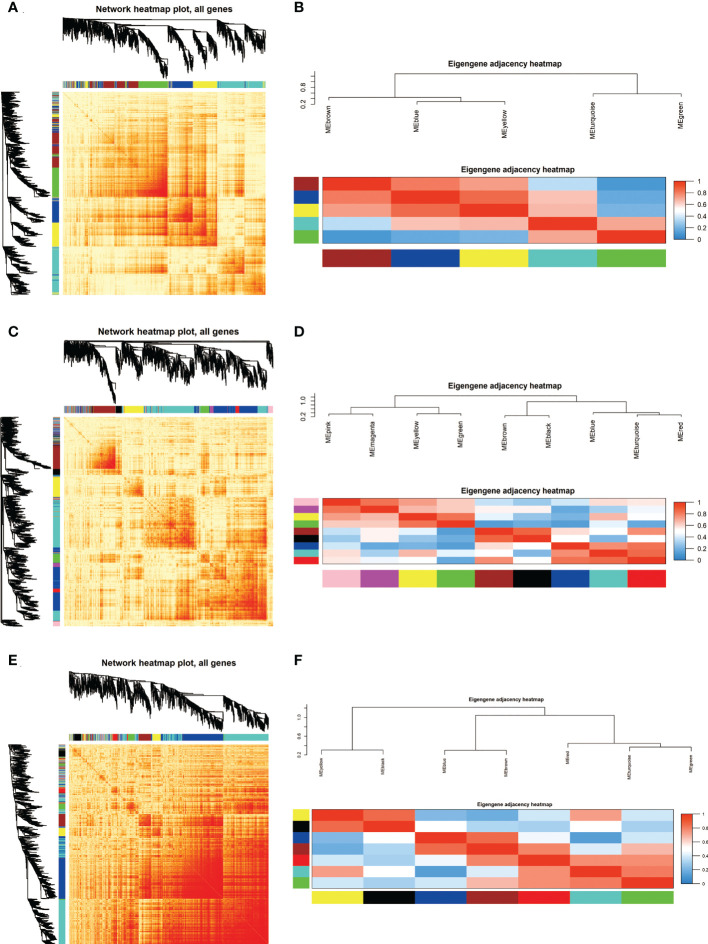
Identification and characterization of autophagy-related genes (ATGs) in Key modules. **(A, C, E)** Co-expression heatmap plot and correlations for ATGs in GSE8157 **(A)**, GSE43264 **(C)** and GSE106724 **(E)** models. The heatmap depicts the Topological Overlap Matrix among all genes in the analysis. The light color represents low overlap and the progressively darker red color represents higher overlap. Blocks of darker colors along the diagonal are the modules. The gene dendrogram and module assignment are also shown along the left side and the top. **(B, D, F)** Clustering of module eigengene to calculate the correlation between each module. The eigengene adjacency heatmap for GSE8157 **(B)**, GSE43264 **(D)**,and GSE106724 **(F)**. Red color represents high adjacency and positive correlation, whereas blue represents low adjacency and negative correlation, as represented by the color legend. The closer the color to Red (to 1), the stronger the correlation.

Based on the co-expressed genes, the heatmap indicated that ATGs in the same module tend to have a high biological significance (**Figures 2A, C, E**). The eigengene dendrogram and heatmap were used to identify groups of correlated eigengenes in modules (**Figures 2B, D, F**). The blue color represents low adjacency (negative correlation), while the red represents high adjacency (positive correlation).

### GO and KEGG enrichment analysis

**Figure 3:**
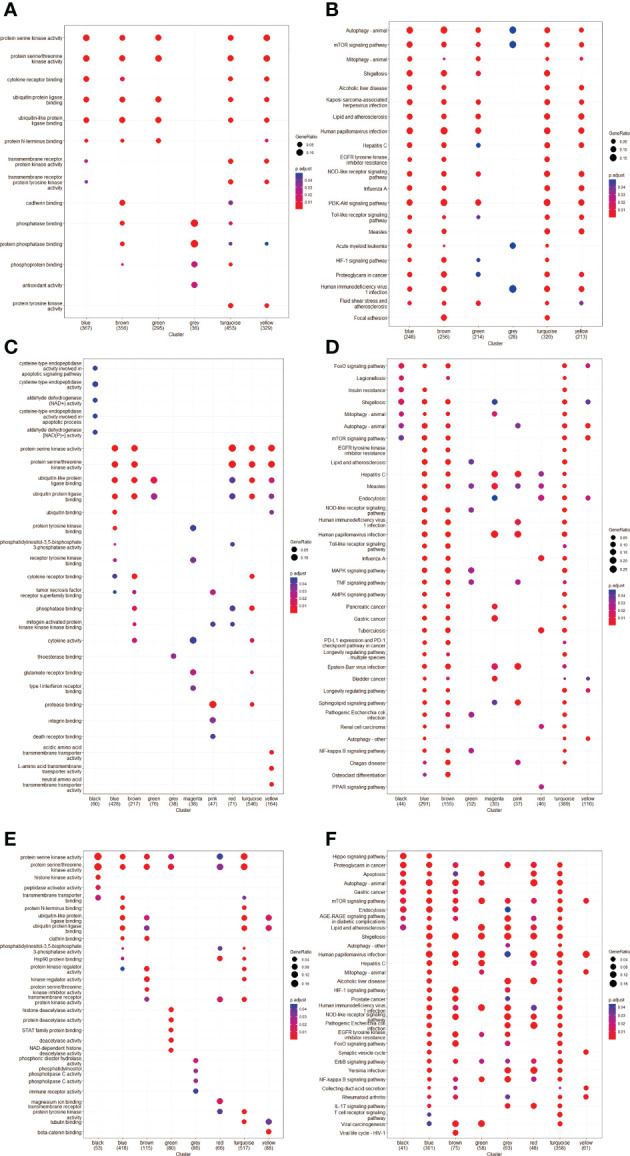
Gene ontology (GO) and Kyoto Encyclopedia of Genes and Genomes (KEGG) pathway enrichment analyses of ATGs in Key modules. **(A, C, E)** The GO enrichment analysis of the modules in GSE8157 **(A)**, GSE43264 **(C)** and GSE106724 **(E)**; X-axis is -log10 of *p*-value and *p* < 0.05 was considered statistically significant. Y-axis shows the biological processes. **(B, D, F)** KEGG **s**ubpathway enrichment analysis of the modules in GSE8157 **(B)**, GSE43264 **(D)** and GSE106724 **(F)**; X-axis is -log10 of *p*-value and *p* < 0.05 was considered statistically significant. Y-axis shows the subpathways.

The GO and KEGG pathway analyses were performed for each module, respectively (**Figure 3**). The GSE8157 datasets primarily focused on autophagy, mitophagy, mechanistic target of rapamycin kinase (mTOR) signaling pathways, and phosphoinositide 3-kinase (PI3K)-serine/threonine kinase (AKT) signaling pathways. Most of the correlated modules were enriched for the regulation of autophagy, macroautophagy, vacuolar membrane, protein serine/threonine kinase activity, and ubiquitin-protein ligase binding (**Figures 3A, B**). Mitogen-activated protein kinase (MAPK) signaling pathway, PI3K-AKT signaling pathways, endocytosis, and autophagy were highly enriched in the GSE43264 datasets. There is an enrichment of regulation of autophagy, macroautophagy, vacuolar membrane, protein serine kinase activity, and ubiquitin-protein ligase binding in Key modules found in the GO pathway (**Figures 3C, D**). It was found that GSE106724 datasets were enriched for autophagy, endocytosis, apoptosis, and mTOR signaling. Most of the correlated ATGs were enriched into the regulation of autophagy, macroautophagy, vacuolar membrane, and protein serine/threonine kinase activity in GO pathway analyses (**Figures 3E, F**). **Supplemental Figure 1** shows the detailed results for each key module.

### Identification and validation of Hub ATGs

**Figure 4:**
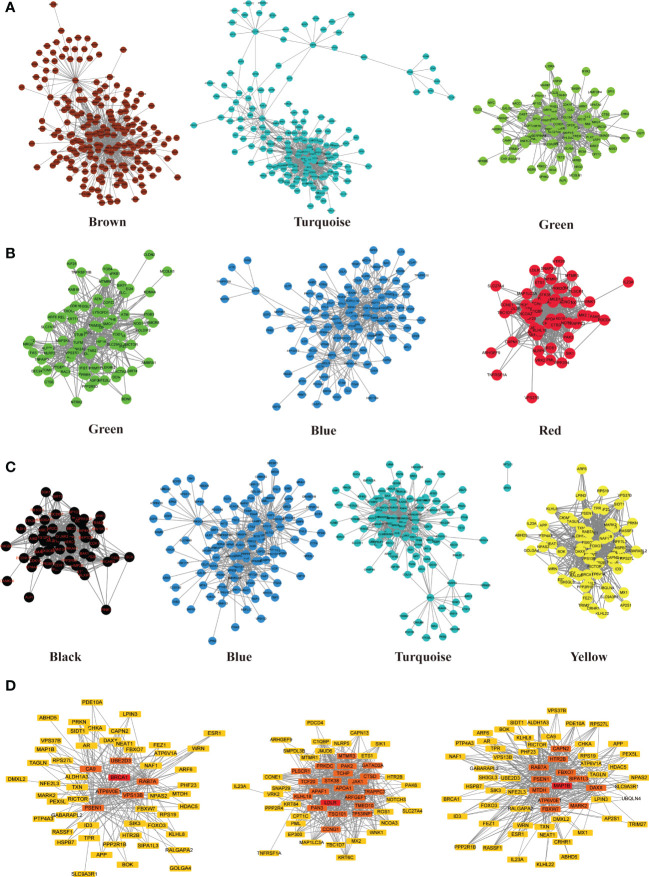
Co-expression network of PCOS-autophagy-related modules and core ATGs visualized by Cytoscape. **(A–C)** Sub-networks of key modules in GSE8157 **(A)**, GSE43264 **(B)** and GSE106724 **(C)**. Module networks extracted from WGCNA. The nodes represent genes. The interaction between each node was evaluated by adding an edge between them. The node’s color is in accordance with the color of the key modules. **(D)** Network analysis with the most correlations identified hub ATGs. The color scale of yellow to Red indicates differences in correlations. The closer the color to Red, the stronger the correlation.

**Figure 5:**
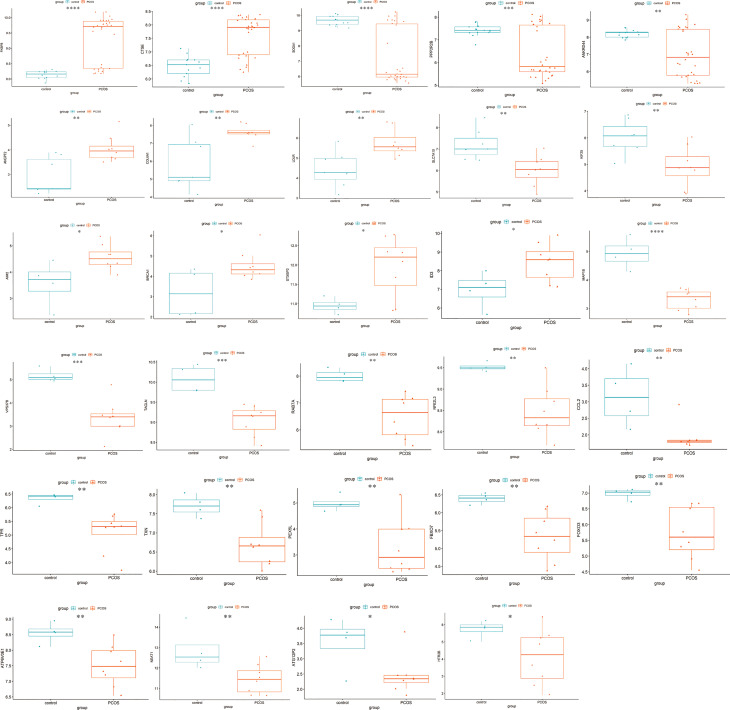
Differential expression of hub ATGs. The box plots showed the differential expression of hub ATGs between women with and without PCOS in GSE51981, GSE43264, and GSE106724. p<0.05 was considered statistically significant. ****: *p* < 0.0001, ***: *p* < 0.001, **: *p* < 0.01, *: *p* < 0.05.

ATGs of key modules were screened and co-expressed gene pairs were retained. The co-expression relationships among the PCOS-autophagy-related modules were analyzed (**Figures 4A–C**). In key modules, the top-ranked ATGs according to the obtained gene significance scores, are centrally located in their respective module and considered to have a greater biological significance. Then, hub ATGs overlapped with the differential expression analysis in datasets, as shown in **Figure 4D**, and the most connected co-expressed genes networks were visualized using Cytoscape. See **Supplemental Table 4** for the Top genes in each module. Thus, GSE8157, GSE43264, and GSE106724 obtained 5, 5, and 19 hub ATGs respectively, and the typical box plot is shown in **Figure 5**. Notably, a significant difference was observed between the PCOS group and the control group in the expression level of each hub ATG.

Moreover, we validated the hub ATGs in the external datasets of GES6798, GSE2508, and GSE95728. **Supplemental Figure 2** shows a comparison of the expression of hub ATGs between each group. These validation results are also roughly consistent with the former findings.

### New molecular subgroups in PCOS

**Figure 6:**
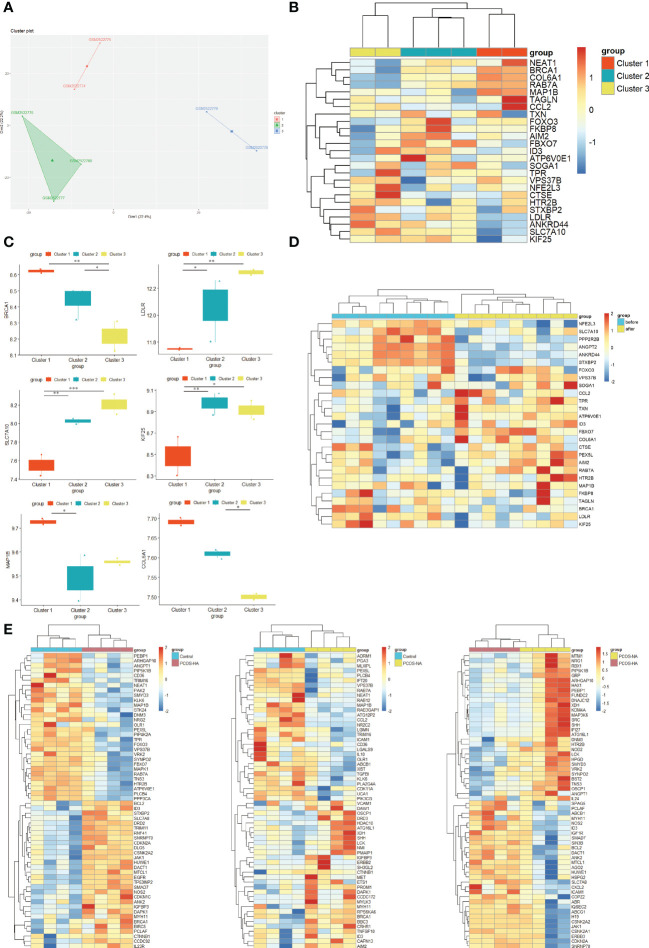
New autophagy-related molecular subgroups in PCOS. **(A)** K-means clustering based on GSE95728. K-means clustering based on SSE to realize the selection of K value (K=3). Tsne diagram shows the clustering of women with PCOS. **(B)** Heatmap showing the expression of 29 co-expressed hub ATGs in PCOS new molecular subgroups. The columns represent 3 subgroups for patients with PCOS (red, blue and yellow columns). The color represents the expression value (log2(exp+1)). Red rows indicate up-regulation, whereas blue rows indicate down-regulation. The darker the color, the greater the difference. **(C)** The expression of hub ATGs in different clusters. Box plot showing the mRNA expression of hub ATGs genes in three clusters. p<0.05 was considered statistically significant. **: *p* < 0.01, *: *p* < 0.05. **(D)** Heatmap showing the expression of the ATGs in PCOS insulin sensitizer treatment groups. The columns represent patients with PCOS before (blue columns) and after pioglitazone treatment (yellow columns). The color represents the expression value (log2(exp+1)). Red rows indicate up-regulation, whereas blue rows indicate down-regulation. The darker the color, the greater the difference. **(E)** Heatmap showing the expression of the ATGs in PCOS-androgen-related subgroups. The columns represent healthy controls (blue columns), PCOS patients with NA(Normal androgen) (yellow columns),and HA(Hyperandrogenism) (red columns). Red rows indicate up-regulation, whereas blue rows indicate down-regulation. The darker the color, the greater the difference.

The optimal K value was three by K-means unsupervised clustering method to classify all PCOS samples in GSE95728. The PCOS patients were appropriately classified into three subgroups when using R tSNE to reduce the dimensionality of gene expression profile data (**Figure 6A**). The hub ATG expression in the different clusters was also analyzed (**Figure 6B**). The combination of all co-expressed hub genes expression is highly consistent with the clustering in **Figure 6A**. Thus, we could speculate that the co-expressed hub ATGs might be necessary for the classification of the PCOS phenotype. A significant difference can be seen in the expression of a certain gene in PCOS subgroups including BRCA1, LDLR, MAP1B, SLC7A10, KIF25, VPS37B, and COL6A1 (**Figure 6C**) (p < 0.05). In subgroups, there was a noticeable trend in the expression levels of ATGs from cluster 1 to cluster 3 (BRCA1, COL6A1, SLC7A10, LDLR). Thus, we speculate these hub ATGs might play a considerably important role as marker genes for PCOS subtypes.

The regulation of autophagy in response to androgen might take a role in PCOS (14). Therefore, we performed the heatmap for ATGs in GSE106724 including PCOS-HA and NA groups (**Figure 6E**). We found that some hub ATGs expression varies with the level of androgen (**Supplemental Figure 3**). The enrichment of ATGs (such as ID3, HTR2B) alters in PCOS-HA and NA groups. The FOXO3, TPR, FBXO7, and STXBP2 expressions were changed in the PCOS-HA groups compared to the control, while the CCL2 and AIM2 expressions were changed in the PCOS-NA groups compared to the control. Thus, the different expressions in ATGs between PCOS-HA, PCOS-NA, and the control groups indicate that there might be a link between autophagy and hyperandrogenism in the PCOS sub-type.

Insulin resistance, a potential pathogenic factor contributing to PCOS, serves as a connecting link between PCOS pathogenesis and metabolic syndrome. A decrease in insulin resistance through insulin-sensitizing drugs (such as metformin, rosiglitazone, pioglitazone, and D-chiro-inositol) could improve the reproductive and metabolic outcomes of patients with PCOS (28, 29). A previous study found that insulin resistance results from the activation of mTOR that attenuates autophagy in PCOS skeletal muscles (18). Thus, Samples of skeletal muscle from GSE8157 datasets including 10 obese women with PCOS before/after pioglitazone treatment were applied to identify the role of autophagy in PCOS-associated insulin resistance. The Heatmap showing the significant differential expression of the hub ATGs in PCOS was partly attenuated by pioglitazone treatment (**Figure 6D**), indicating a greater role in PCOS with insulin resistance.

**Figure 7:**
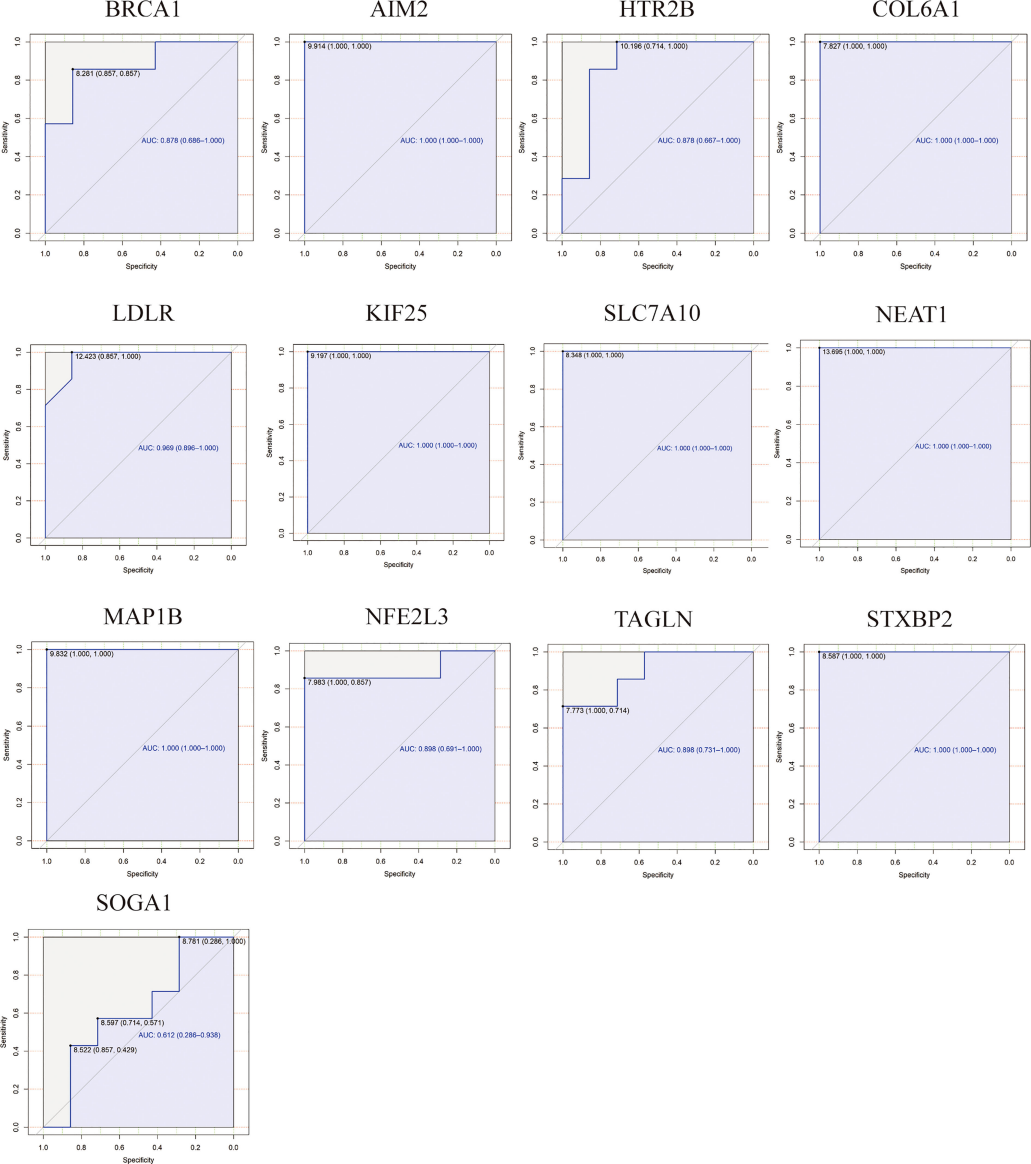
Receiver Operating Characteristic (ROC) and area under the curve (AUC) values analysis for hub ATGs. The specificity and sensitivity of the hub ATGs for the diagnosis of PCOS by the ROC analysis. AUC values exceeding 60% are considered to be acceptable, with 90% indicating higher sensitivity and specificity.

The ROC analysis was employed to evaluate the specificity and sensitivity of the hub ATGs for the diagnosis of PCOS (**Figure 7**). GSE95728 showed AUC values exceeding 60% considered to be acceptable, for 12 hub ATGs. For AIM2, COL6A1, LDLR, KIF25, SLC7A10, NEAT1, MAP1B, and STXBP2, AUC values exceeded 90%, indicating higher sensitivity and specificity.

### Expression profiles of PCOS transcripts

**Figure 8:**
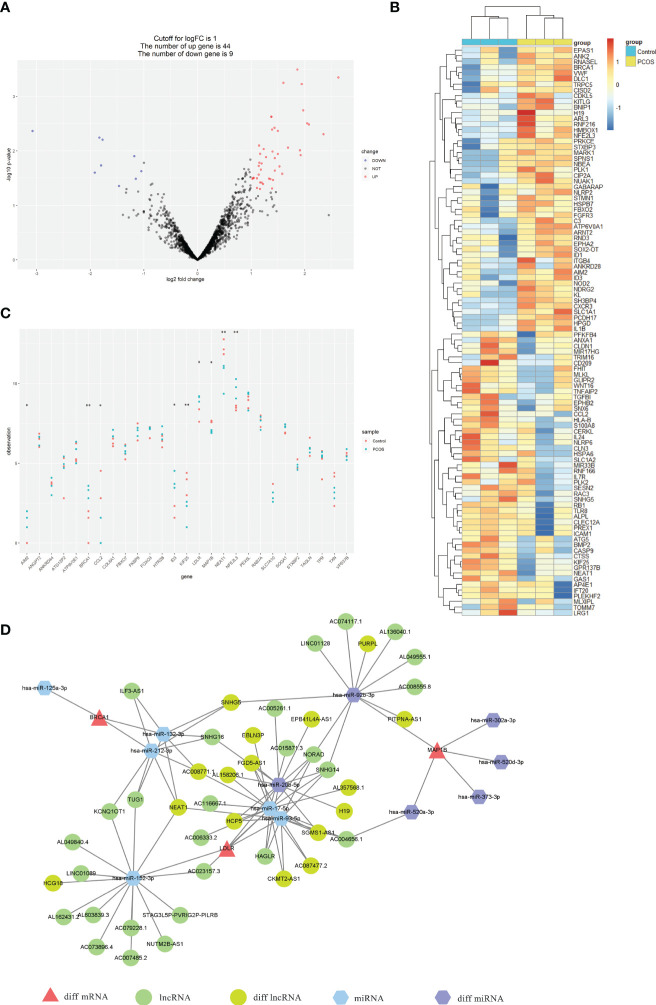
Expression profiles of ATGs by RNA-seq and construction of the multifactorial PCOS-autophagy-related network. **(A)** Volcano plots visualized RNA-seq results showing differential expression ATGs. The differences between the groups are plotted on the X-axis. The log10(p-value) for the differences is plotted on the Y-axis. **(B)** Heatmap showing the differential expression ATGs by RNA-seq. The columns represent healthy controls (blue columns) and PCOS patients(yellow columns). Red rows indicate up-regulation, whereas blue rows indicate down-regulation. The darker the color, the greater the difference. **(C)** Scatter plot showing the expression of hub ATGs *via* RNA-seq between healthy controls (red node) and PCOS patients (blue node). p<0.05 was considered statistically significant. **: *p* < 0.01, *: *p* < 0.05. **(D)** The mRNA-miRNA-lncRNA multifactorial network visualized by Cytoscape consists of 3 mRNAs (red triangles), 6 miRNAs (blue and purple hexagons), and 16 lncRNAs (green ellipses).

To identify DEGs that are potentially involved in the etiology of PCOS, we evaluated transcripts expression profiles in ovarian granulosa cells by RNA-seq. A total of 765 genes exhibited differential expression between women with and without PCOS (**Supplemental Figure 4A**). The heatmap of the top 50 DEGs is shown in **Supplemental Figure 4B**. Among those genes, 53 were ATGs, 44 were up-regulated and 5 were down-regulated (**Figure 8A**). The heatmap of the top 50 ATGs is reported in **Figure 8B**. Notably, these DEGs between PCOS and health control in accordance with the hub ATGs we identified before, such as BRCA1, KIF25, NEAT1, NFE2L3, LDLR, AIM2, CCL2, MAP1B, and ID3 (**Figure 8C**). This suggests that these hub ATGs might be closely related to PCOS dysfunction. The expression of hub ATGs is provided in **Supplemental Table 5**. The RNA-seq has been uploaded to the NGDC database (BioProject ID: PRJCA012180).

### Construction of PCOS-autophagy-related multifactorial network

A total of 12 miRNA-hub ATGs were acquired from the miRwalk 3.0 database according to the interaction relationship in 3′ UTR, 5’ UTR, and CDS regions. Besides, 43 miRNA-lncRNA interaction relationships were obtained based on the StarBase v3.0 database (**Supplemental Table 6**). The interaction between each node was evaluated by adding an edge between them. The analysis of variance resulted in 16 lncRNA and 6 miRNA that overlapped between the above-mentioned analysis results and network relationships (**Supplemental Table 6**). The nodes shared with more connected edges and differentially expressed in the network are considered to be more important. Thus, 3 mRNAs (BRCA1, LDLR, and MAP1B), 2 miRNAs (hsa-miR-92b-3p and hsa-miR-20b-5p), and 1 lncRNA (NEAT1) were identified as the key factors in the PCOS-autophagy-related network. (**Supplemental Table 7**). Finally, we successfully constructed the mRNA-miRNA-lncRNA multifactorial network, as shown in **Figure 8D**.

## Discussion

Autophagy had been reported to be associated with significant abnormalities such as anovulation, hyperandrogenism, and metabolic disturbances in PCOS. However, detailed research in this area remains limited. A major objective of our study is to discover whether autophagy is related to PCOS, especially in the ovary, adipose tissue, and skeletal muscles. In this study, the ATGs and new molecular subgroups associated with PCOS were identified, and a multifactorial network was constructed. The co-expression network was applied to investigate the potential molecular mechanisms and biological processes for the pathogenesis as well as subtype of PCOS. We, therefore, proposed a novel proposal for future studies to better understand PCOS pathogenesis.

Previous studies have tried to interpret the molecular mechanisms of PCOS *via* a single microarray dataset, leading to a relatively high possibility of false-positive results. Thus, Our work analyzed a series of mRNA expression profile datasets from different tissue to explore the association between ATGs and anovulation, folliculogenesis, and metabolic disturbances in PCOS. The crucial ATGs were identified by WGCNA *via* GSE106724, GSE43264, and GSE8157. The external datasets, GES6798, GSE2508, GSE95728, and GSE72274 were applied to the validate results and reveal new molecular subgroups in the PCOS phenotyping.

We found the GO and KEGG analyses of key modules in WGCNA to be mainly associated with autophagy-related functions and pathways. The processes utilizing the autophagic mechanism were highly enriched in the key module *via* KEGG analyses such as autophagy, endocytosis, apoptosis, mTOR signaling pathways, MAPK signaling pathway, and PI3K-AKT signaling pathways. The GO analyses were focused on processes including autophagy, vacuolar membrane, protein serine kinase activity, protein serine/threonine kinase activity, and ubiquitin-protein ligase binding. GO and KEGG analysis results further confirmed the involvement of ATGs in the regulation of certain signaling pathways in PCOS.

An altered expression of autophagic genes is associated with PCOS pathogenesis (30), is observed in particular cellular mechanisms, and is associated with signaling pathways, such as mTOR, MAPK, AKT, and FoxO. Various studies have reported that AKT-mediated mTOR-autophagy axis is involved in regulating ovarian folliculogenesis and follicular development (12, 31, 32). The inhibition of the PI3K-AKT-MTOR pathway induces autophagy accompanied by apoptosis, resulting in massive destruction of granulosa cells, which then triggers follicular atresia (12). Other studies demonstrated the involvement of the activation of the mTOR pathway inhibits autophagy and promotes aberrant growth of follicles (33, 34). In addition, FoxO signaling pathways was enriched in GSE43264. It has been reported that PCOS is also associated with PI3K-AKT-FOXO1-MTOR signaling (35). Therefore, we suggest that the autophagic impairment and mechanisms in PCOS might be caused by an alternation of ATGs related to certain autophagy functions and signaling pathways.

In key modules, we screened 29 different expressed ATGs in women with PCOS. Some of these ATGs were already identified in previous studies. BRCA1, a hub gene in the co-expression relationships network, was a significant increase in key modules and was validated in an external dataset. BRCA1, a tumor suppressor associated with the development of breast, and ovarian, participate in multiple biological pathways including the DNA damage response, transcriptional control, cell growth, and apoptosis (36). BRCA1-related ataxia telangiectasia mutated-mediated DNA double-stranded breaks repair function emerges as a likely regulator of age in human oocytes (37). In women with PCOS, there is a significant increase in frequencies of BRCA1 polymorphisms genotypes and alleles in patients compared to controls (38, 39). As well as serving as a key gene in the PCOS-autophagy-related network, LDLR is altered in the PCOS group. A prenatal androgen exposure could contribute to PCOS by affecting reproductive functions. LDLR levels were elevated in anovulatory prenatally hyperandrogenized rats, along with altered lipid profiles, hormone profiles, and steroidogenesis, as well as altered ovarian lipid metabolism (40). PCSK9/LDL-r/LDL might be involved in the development and progression of PCOS (41). MAP1B, critically important in the localization of specific RNAs and maintenance of cell shape, is a major neuronal cytoskeleton protein that interacts with microtubules of the cellular cytoskeleton. Down-regulation of MAP1B was found in follicular cystic follicles in bovine ovaries (42). NEAT1, known as a key regulator for many diseases, also functions as an ATG in the PCOS-autophagy-related network. NEAT1 interference-mediated effect induces proliferation and represses apoptosis of ovarian granulosa cells in polycystic ovary syndrome *via* the microRNA-381/IGF1 axis (43).

Molecular subtypes have been defined in various diseases such as cancers and autoimmune disorders. To enable personalized treatment for PCOS, it is essential to determine the molecular subtypes of the disease and how they correlate with clinical characteristics. The clustering analysis of the GSE95728 datasets using K-means further classified the PCOS patient into three new molecular subgroups. Although there was no difference in the expression of some genes in our classification, Accordingly, we observed differences in significant expression levels of ATGs across clusters including BRCA1, LDLR, MAP1B, SLC7A10, KIF25, VPS37B, and COL6A1. In addition, a variation trend in the expression levels of ATGs was found. The expression level of LDLR and SLC7A10 shared a similar upward trend from cluster 1 to cluster 3, whereas BRCA1 and COL6A1 showed a downward trend. The ROC curve of hub genes suggests high sensitivity and specificity. Hence, co-expressed hub ATGs might contribute to the classification of PCOS phenotype.

PCOS is both an endocrine and metabolic disorder. The altered ATGs may exhibit different clinical characteristics in PCOS. Hyperandrogenism is one of the characteristic endocrine features of PCOS. The autophagy regulation of androgen might be reponed to PCOS pathogenesis. We found that the ATGs were expressed differently between PCOS-HA, NA groups, and controls (**Figure 6E**),including ID3, HTR2B, FOXO3, TPR, FBXO7, STXBP2, CCL2, and AIM2. It has been reported that PCOS has a different autophagic response due to an increase in androgen. In skeletal muscles, testosterone activates MTORC1, which inhibits autophagy proteins (18). The uterus exhibits altered gene expression in response to hyperandrogenism, inhibiting autophagy (44, 45). In granulosa cells, testosterone promotes autophagy by inducing the expression of BECN1, ATG, and LC3 (13, 46). Immunohistochemistry of a PCOS-affected ovary shows the accumulation of Sequestosome 1 and ubiquitin in the Theca cells (TCs) layer. Autophagy is suppressed in TCs, which can lead to ROS production (47).

The role of autophagy in various PCOS-associated metabolic abnormalities such as insulin resistance and abnormal adiposity acquired in various metabolic organs. To explore the metabolic function of ATGs in PCOS, we utilized samples of adipose tissue and skeletal muscles as metabolic organs. The pathway analyses found that insulin resistance was enriched in GSE43264, whereas lipid and atherosclerosis, and alcoholic liver disease were enriched in both GSE8157 and GSE106724. Metabolic genes such as LDLR were identified as hub ATGs in key modules. We also found the hub ATGs expression were partly attenuated in PCOS *via* pioglitazone treatment (**Figure 6D**). Moreover, we extracted genes involved in adipokines and insulin receptor proteins from databases including GeneCards and NCBI. The volcano plots and heatmap plots showed the variation of the differential RNA expressions in the valuable datasets *via* the R Package (**Supplemental Figure 5**, **6**). The BRCA1 (P = 0.018) and COL6A1 (P = 0.002) were enrichment in both adipokines and insulin signaling pathway; the LDLR (P = 0.004) and TAGLN (P < 0.001) expressed differently in adipokines pathway while the MAP1B (P < 0.001), NEAT1 (P = 0.008), ANGPT2 (P = 0.002), ID3 (P = 0.017), PEX5L (P = 0.005) and RAB7A (P = 0.001) were differently expressed in insulin signaling pathway (**Supplemental Table 8**). The results indicate that ATGs might play a crucial role in metabolic abnormalities such as adipocyte dysfunction and insulin resistance during PCOS progression. In PCOS, autophagy is vital to the metabolic response in affected tissues. The induction of autophagy suppresses obesity-driven insulin resistance due to adipocyte dysfunction and defaulted insulin signaling pathways in adipose tissue (16, 48). There is a direct relationship between hyperinsulinemia and hyperandrogenism in the ovary, where insulin promotes the expression of steroidogenic enzymes ARK1C3 and CYP17A1 (49). Insulin resistance and hyperandrogenism can be inhibited by regulating autophagy. In PCOS skeletal muscles, insulin resistance is attributed to the activation of mTOR that attenuates autophagy (18). Additionally, a correlation exists between PCOS and damage associated with autophagy in the hepatocytes of patients suffering from nonalcoholic steatohepatitis (50).

In this work, the RNA-seq was used to identify the DEGs and further validate hub ATGs expression between women with PCOS and health control. A multifunctional co-expression network was constructed among mRNA, miRNA, and lncRNA to further explore the mechanism of PCOS. Mapping the DEGs into the modules, 4 of the hub ATGs (BRCA1, LDLR, MAP1B, and NEAT1) were selected to construct the lncRNA-miRNA-mRNA network. Differentially expressed miRNAs and lncRNAs overlapped with GSE72274 and GSE95728. The genes from the multifunctional network were tightly linked and cross-regulated with each other. Some edges were already reported in other diseases. Dysregulation of the BRCA1/NEAT1 axis might contribute to breast tumorigenesis (51). The NEAT1/miR−204/BRCA1/BRCA2 complex subunit 3 axis protects endothelial cells against hypoxia/reoxygenation−induced NLRP3 inflammasome activation (52). It has been reported that BRCA1/hsa-miR-212 axis plays a potential role in the radiotherapy of gliomas by regulating apoptosis (53). NEAT1-miR-212-3p-TTK is reported to be a potential RNA regulatory pathway that controls disease progression in early Rheumatoid Arthritis (54). NEAT1 regulation of the miR-212-3p/AXIN1 pathway might be a therapeutic target for neuroprotection in Parkinson’s Disease (55). NEAT1 promoted glioma progression *via* miR-152-3p/CCT6A and miR-132/SOX2 pathway (56, 57). NEAT1 regulating miR-93-5p/TXNIP axis thus promotes lipopolysaccharide-induced injury (58). NEAT1/miR-17-5p/TGFβR2 axis promotes gastric cancer progression through up-regulated angiogenesis (59). Nevertheless, the detailed mechanism of the multifunctional PCOS-autophagy-related network needs to be studied further in the future.

The limitation in this study mainly lies in the limitation of samples. There may have been heterogeneity in the analysis because the data were collected from different tissues or cells as well as different phenotypes. **Supplemental Table 1** provides the patient’s clinical information from different datasets and RNA-seq. However, due to the limitations of the GEO database, the raw clinical data of some patients cannot be fully obtained. Currently, clinical samples have not been validated and a further experiment is required to verify these results. Still, our results will provide a valuable contribution to future research endeavors in this field.

In conclusion, multifactorial PCOS-autophagy-related networks have been successfully constructed in this work. BRCA1, LDLR, MAP1B, and NEAT1 might be effective diagnostic biomarkers for PCOS. The three new molecular subgroups of PCOS presented in this study based on hub ATGs would assist to adapt the intervention. The three molecular subgroups of PCOS were identified based on hub ATGs, allowing the intervention to be tailored. The hub ATGs might play a vital role in endocrine and metabolic abnormalities such as hyperandrogenism, insulin resistance, and adipocyte dysfunction during PCOS progression. The above-mentioned results could be useful to investigate PCOS pathogenesis and discover novel biomarkers and therapeutic targets. Further research plans include the investigation of the mechanism of action of ATGs and the collection of larger clinical samples.

## Data availability statement

The RNA-seq profile of 6 samples (3 PCOS and 3 health control) is available at the NGDC database (https://ngdc.cncb.ac.cn/), BioProject ID: PRJCA012180.

## Ethics statement

The studies involving human participants were reviewed and approved by The Ethics committee of the Seventh Affiliated Hospital of Sun Yat-sen University (KY-2022-075-01). The patients/participants provided their written informed consent to participate in this study.

## Author contributions

JH authored this manuscript. JH and LM contributed conception of the study; JH designed the study. JH, BH, and YK organized the database and performed the statistical analysis; JH wrote the first draft of the manuscript; YY, LC, CT, and YL revised the manuscript. All authors contributed to the article and approved the submitted version.

## Conflict of interest

The authors declare that the research was conducted in the absence of any commercial or financial relationships that could be construed as a potential conflict of interest.

## Publisher’s note

All claims expressed in this article are solely those of the authors and do not necessarily represent those of their affiliated organizations, or those of the publisher, the editors and the reviewers. Any product that may be evaluated in this article, or claim that may be made by its manufacturer, is not guaranteed or endorsed by the publisher.

## Supplementary material

Supplementary Figure 1 GO and KEGG pathway enrichment analyses of each module in GSE8157 **(A)**, GSE43264 **(B)** and GSE106724 **(C)**.

Supplementary Figure 2 The expression of hub ATGs were validated in the three external datasets of GES6798, GSE2508 and GSE95728.

Supplementary Figure 3 The hub ATGs expression varies with the level of androgen in GSE106724

Supplementary Figure 4
**(A)** Volcano plots visualized RNA-seq results showing differential expression genes (DEGs). The differences between the groups are plotted on the X-axis. The log10(p-value) for the differences are plotted on the Y-axis. **(B)** Heatmap showing the DEGs by RNA-seq. The columns represent healthy controls (blue columns) and PCOS patients(yellow columns). Red rows indicate up-regulation, whereas blue rows indicate down-regulation. The darker the color, the greater the difference.

Supplementary Figure 5
**(A, C, D)** Heatmap showing the DEGs related to adipokines by RNA-seq in GSE8157 **(A)**, GSE43264 **(C)** and GSE106724 **(E)**. The columns represent healthy controls(blue columns) and PCOS patients(yellow columns). Red rows indicate up-regulation, whereas blue rows indicate down-regulation. The darker the color, the greater the difference. **(B, D, F)** Volcano plots visualized RNA-seq results showing DEGs related to adipokines in GSE8157 **(B)**, GSE43264 **(D)** and GSE106724 **(F)**. The differences between the groups are plotted on the X-axis. The log10(p-value) for the differences are plotted on the Y-axis.

Supplementary Figure 6
**(A, C, D)** Heatmap showing the DEGs related to insulin signaling by RNA-seq in GSE8157 **(A)**, GSE43264 **(C)** and GSE106724 **(E)**. The columns represent healthy controls (blue columns) and PCOS patients(yellow columns). Red rows indicate up-regulation, whereas blue rows indicate down-regulation. The darker the color, the greater the difference. **(B, D, F)** Volcano plots visualized RNA-seq results showing DEGs related to insulin signaling in GSE8157 **(B)**, GSE43264 **(D)** and GSE106724 **(F)**. The differences between the groups are plotted on the X-axis. The log10(p-value) for the differences are plotted on the Y-axis.
