# Supplementary figures and images for: Polycystic ovary syndrome: Identification of novel and hub biomarkers in the autophagy-associated mRNA-miRNA-lncRNA network
Source: Front Endocrinol (Lausanne). 2022 Nov 29;13:1032064. doi: 10.3389/fendo.2022.1032064 (PMC9745174; doi:10.3389/fendo.2022.1032064)

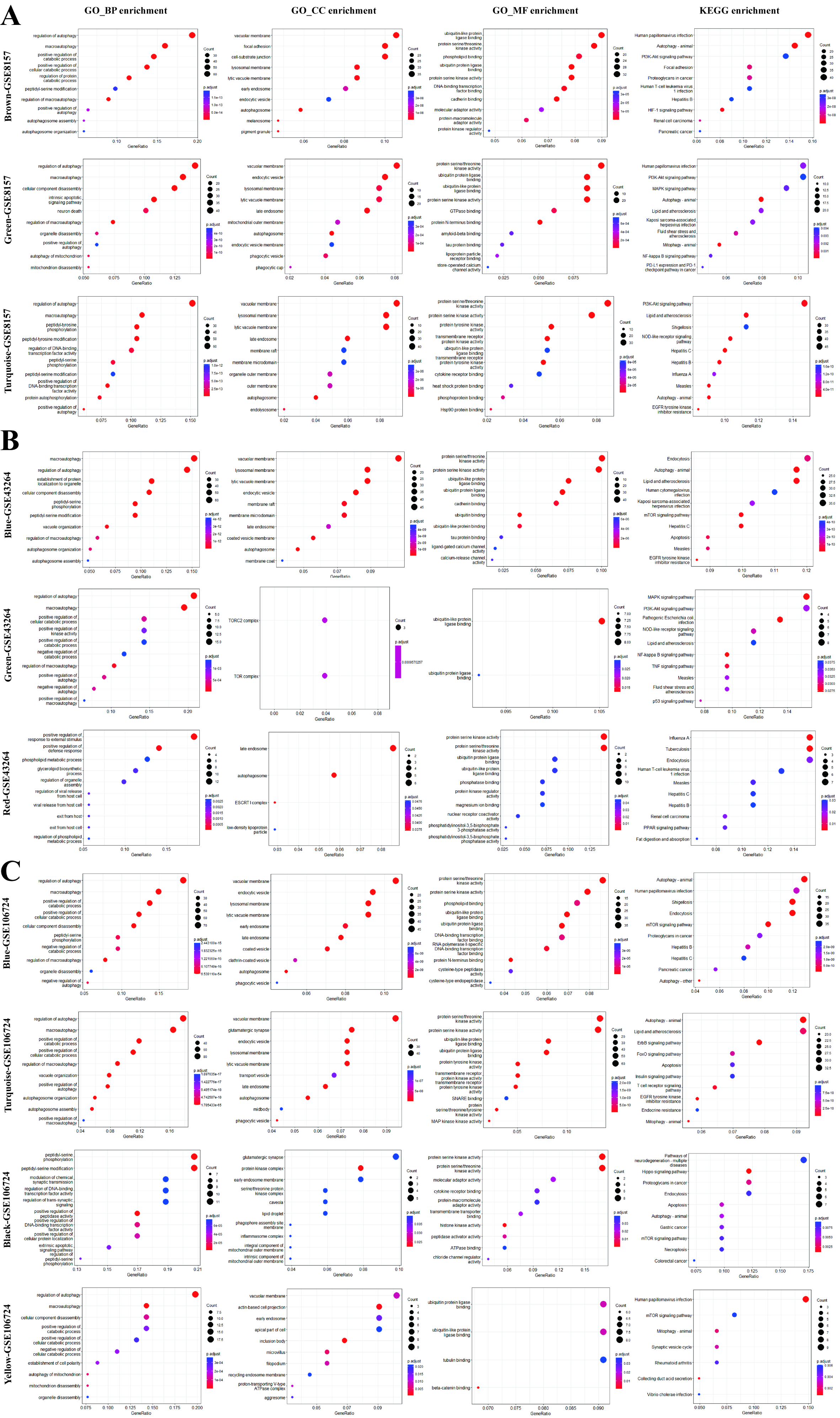

Supplement: Supplementary Figure 1 — GO and KEGG pathway enrichment analyses of each module in GSE8157 (A), GSE43264 (B) and GSE106724 (C). [file Image_1.tif]

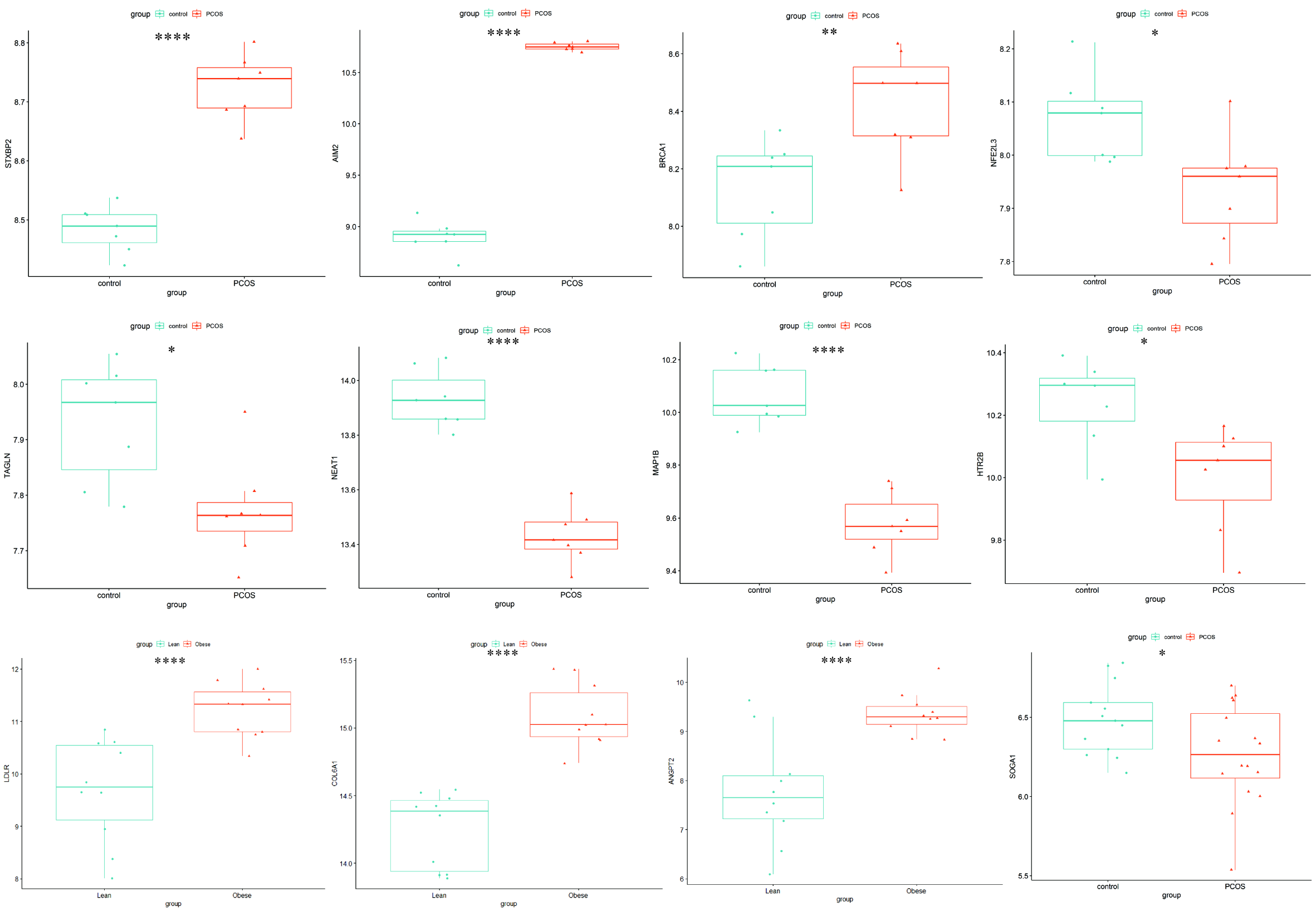

Supplement: Supplementary Figure 2 — The expression of hub ATGs were validated in the three external datasets of GES6798, GSE2508 and GSE95728. [file Image_2.tif]

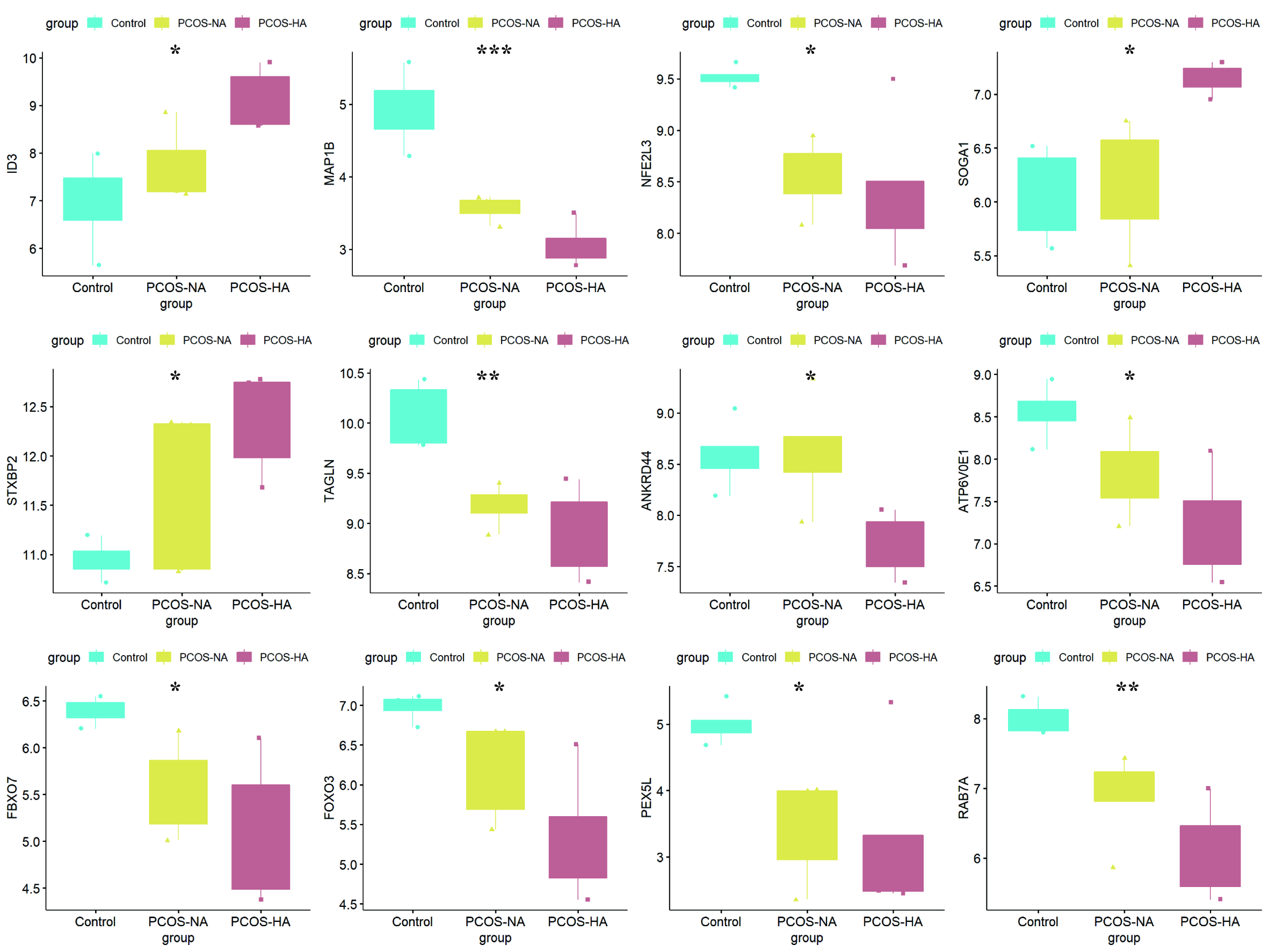

Supplement: Supplementary Figure 3 — The hub ATGs expression varies with the level of androgen in GSE106724 [file Image_3.tif]

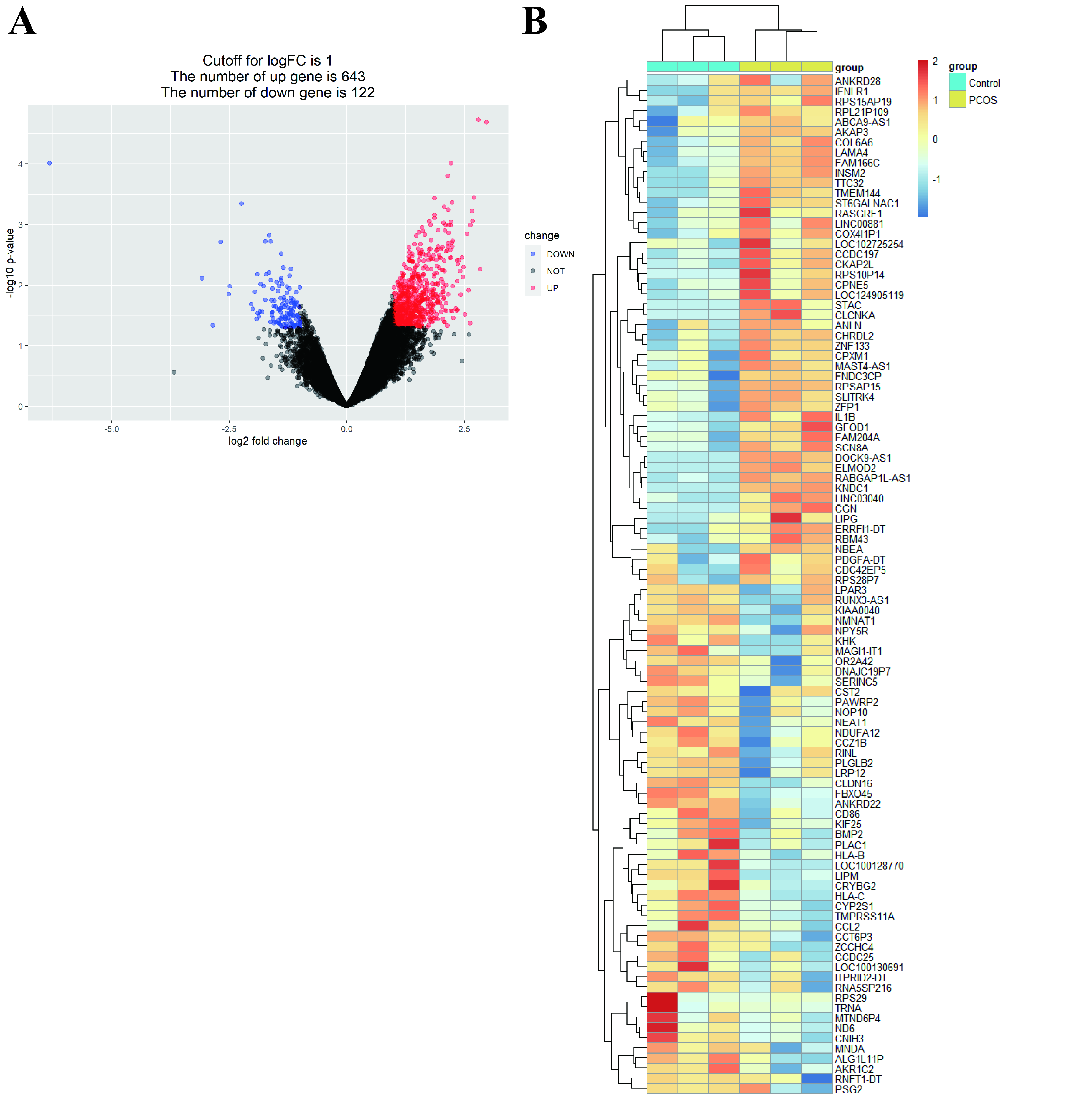

Supplement: Supplementary Figure 4 — (A) Volcano plots visualized RNA-seq results showing differential expression genes (DEGs). The differences between the groups are plotted on the X-axis. The log10(p-value) for the differences are plotted on the Y-axis. (B) Heatmap showing the DEGs by RNA-seq. The columns represent healthy controls (blue columns) and PCOS patients(yellow columns). Red rows indicate up-regulation, whereas blue rows indicate down-regulation. The darker the color, the greater the difference. [file Image_4.tif]

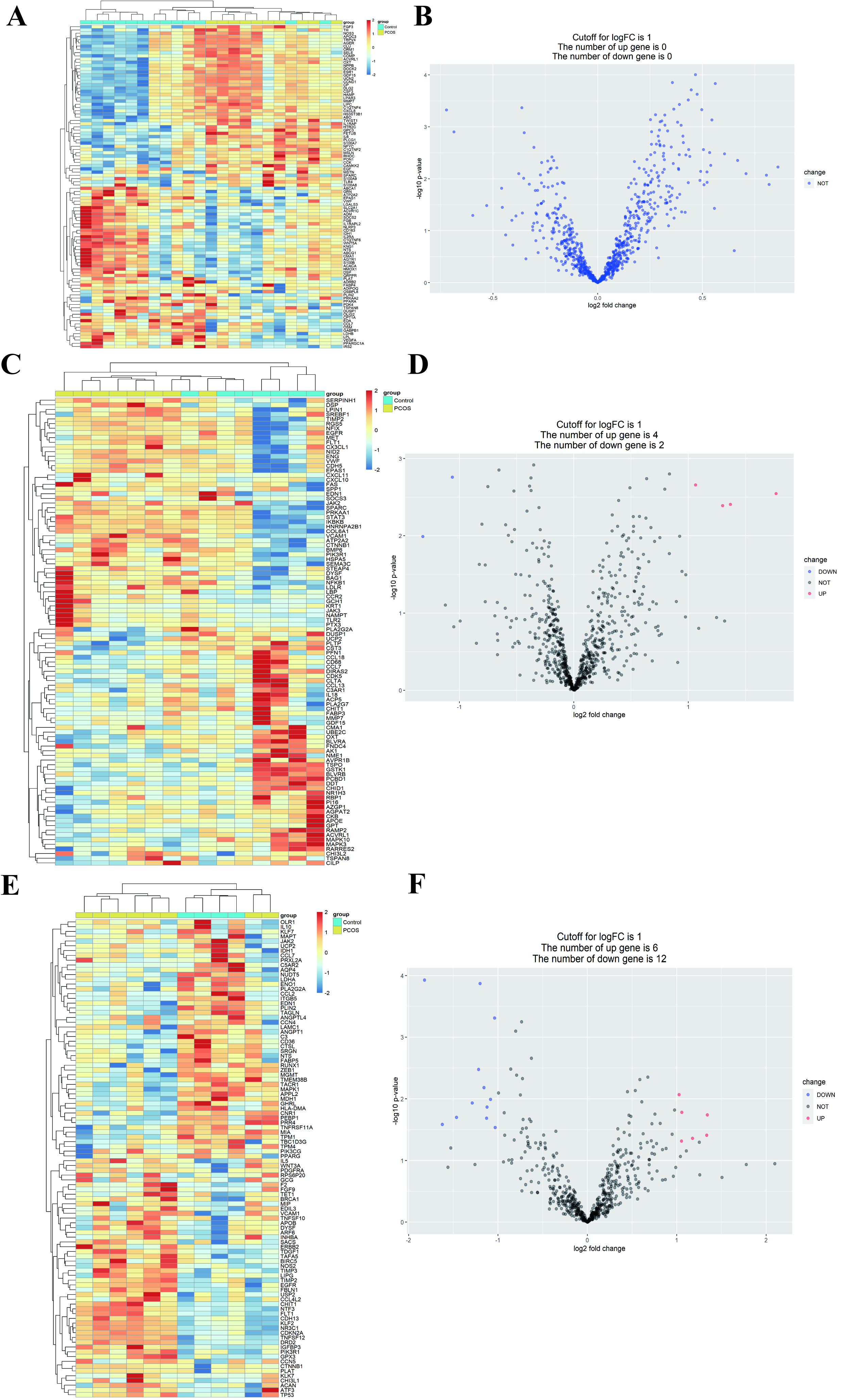

Supplement: Supplementary Figure 5 — (A, C, D) Heatmap showing the DEGs related to adipokines by RNA-seq in GSE8157 (A), GSE43264 (C) and GSE106724 (E). The columns represent healthy controls(blue columns) and PCOS patients(yellow columns). Red rows indicate up-regulation, whereas blue rows indicate down-regulation. The darker the color, the greater the difference. (B, D, F) Volcano plots visualized RNA-seq results showing DEGs related to adipokines in GSE8157 (B), GSE43264 (D) and GSE106724 (F). The differences between the groups are plotted on the X-axis. The log10(p-value) for the differences are plotted on the Y-axis. [file Image_5.tif]

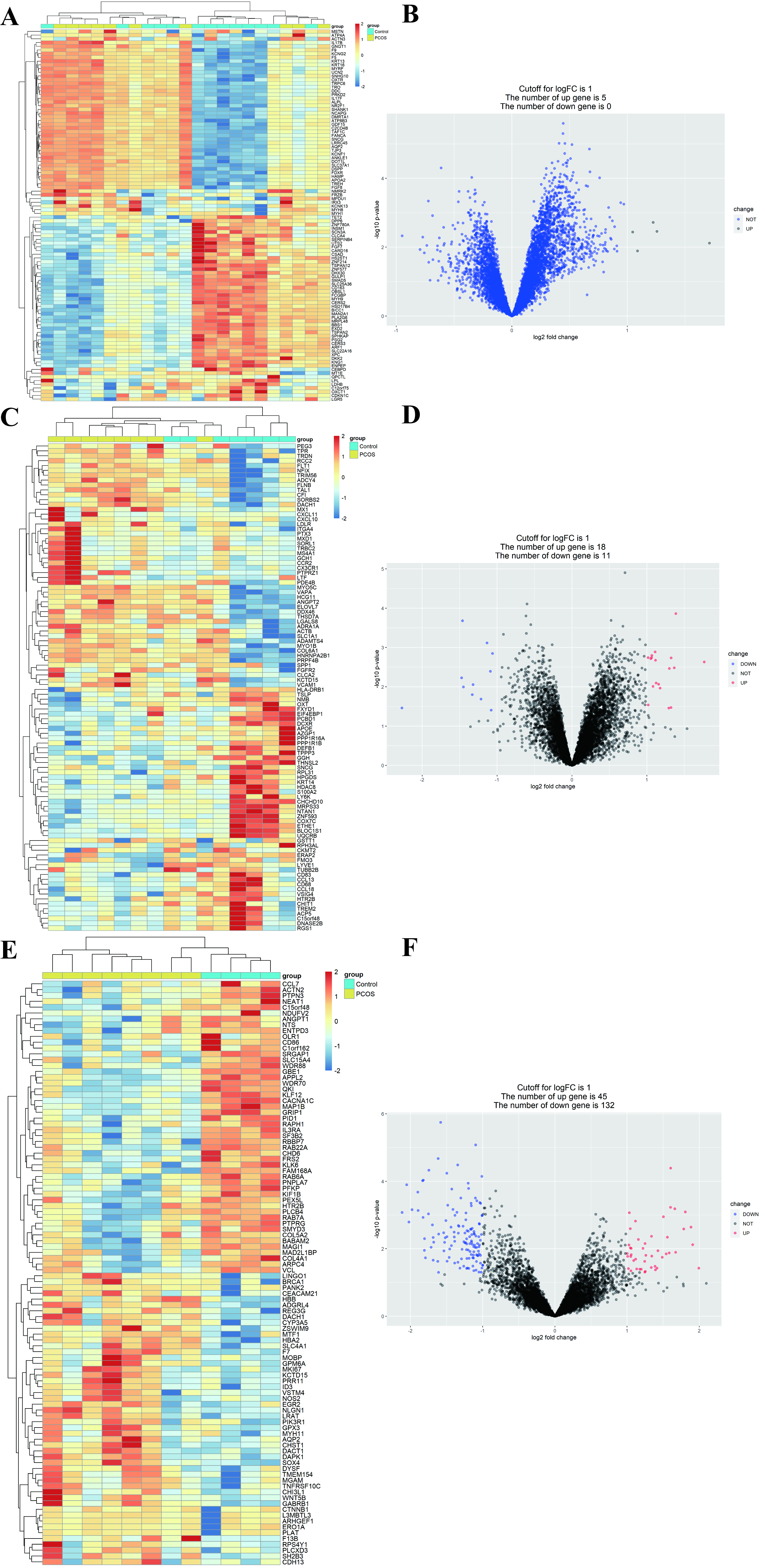

Supplement: Supplementary Figure 6 — (A, C, D) Heatmap showing the DEGs related to insulin signaling by RNA-seq in GSE8157 (A), GSE43264 (C) and GSE106724 (E). The columns represent healthy controls (blue columns) and PCOS patients(yellow columns). Red rows indicate up-regulation, whereas blue rows indicate down-regulation. The darker the color, the greater the difference. (B, D, F) Volcano plots visualized RNA-seq results showing DEGs related to insulin signaling in GSE8157 (B), GSE43264 (D) and GSE106724 (F). The differences between the groups are plotted on the X-axis. The log10(p-value) for the differences are plotted on the Y-axis. [file Image_6.tif]
